# Efficacy of crizotinib retreatment after crizotinib-related interstitial lung disease in a patient with ROS1-rearranged advanced lung adenocarcinoma: A case report and potential crizotinib retreatment strategy

**DOI:** 10.3389/fonc.2022.900966

**Published:** 2022-10-18

**Authors:** Woo Kyung Ryu, Hyungkeun Cha, Mi Hwa Park, Jung Soo Kim, Jeong-Seok Choi, Lucia Kim, Kyung-Hee Lee, Hae-Seong Nam

**Affiliations:** ^1^ Division of Pulmonology, Department of Internal Medicine, Inha University Hospital, Inha University School of Medicine, Incheon, South Korea; ^2^ Department of Otorhinolaryngology-Head and Neck Surgery, Inha University Hospital, Inha University School of Medicine, Incheon, South Korea; ^3^ Department of Pathology, Inha University Hospital, Inha University School of Medicine, Incheon, South Korea; ^4^ Department of Radiology, Inha University Hospital, Inha University School of Medicine, Incheon, South Korea

**Keywords:** interstitial lung disease (ILD), ALK (anaplastic lymphoma kinase), ROS1, tyrosine kinase inhibitor (TKI), crizotinib

## Abstract

Crizotinib is an oral selective small-molecular tyrosine kinase inhibitor (TKI) that suppress the activity of anaplastic lymphoma kinase (ALK) and ROS1 kinases, as well as mesenchymal-epithelial transition. The cumulative clinical trials in patients with advanced ALK- or ROS1-rearrangement NSCLC indicate that crizotinib has significant antitumor activity and a tolerable safety profile, with mild or moderate adverse events of visual disorders, diarrhea, nausea, and vomiting. As with other TKIs, however, the occurrence of crizotinib-related interstitial lung disease (crizotinib-ILD) remains a major clinical dilemma that can lead to the permanent discontinuation of TKI during cancer treatment. When there is no suitable alternative therapy for patients who develop crizotinib-ILD, some clinicians have reported successful crizotinib retreatment in cases of ALK-rearrangement NSCLC. Unfortunately, there are no specific guidelines for the treatment or retreatment of TKI-related ILD. We herein report the first successful crizotinib retreatment after crizotinib-ILD in a patient with ROS1-rearranged NSCLC, and suggest a retreatment strategy after crizotinib-ILD based on a literature review.

## Introduction

ROS1 proto-oncogene receptor tyrosine kinase is activated by chromosomal rearrangement in a variety of human cancers, including non-small cell lung cancer (NSCLC) (1). ROS1-rearrangements occur in approximately 1−2% of patients with NSCLC and are more common in those who have no or only a light smoking history and NSCLC of adenocarcinoma histology (1–3). Crizotinib is the only oral tyrosine kinase inhibitor (TKI) approved by the US Food and Drug Administration (FDA) (in March 2016) for patients with advanced ROS1-rearrangement NSCLC (4).

Although crizotinib has clear clinical benefits and has led to a paradigm shift in the treatment of advanced ROS1-rearrangement NSCLC (1, 2), the occurrence of crizotinib-related interstitial lung disease (crizotinib-ILD) remains a major clinical dilemma that can lead to the permanent discontinuation of TKI during cancer treatment. When there is no suitable alternative therapy for patients who develop crizotinib-ILD, some clinicians have reported successful crizotinib retreatment in cases of anaplastic lymphoma kinase (ALK)-rearrangement NSCLC (5–9). However, there are no specific guidelines for the treatment or retreatment of TKI-related ILD. We herein report the first successful crizotinib retreatment after crizotinib-ILD in a patient with ROS1-rearranged NSCLC, and suggest a retreatment strategy after crizotinib-ILD based on a literature review

## Case presentation

**Figure 1:**
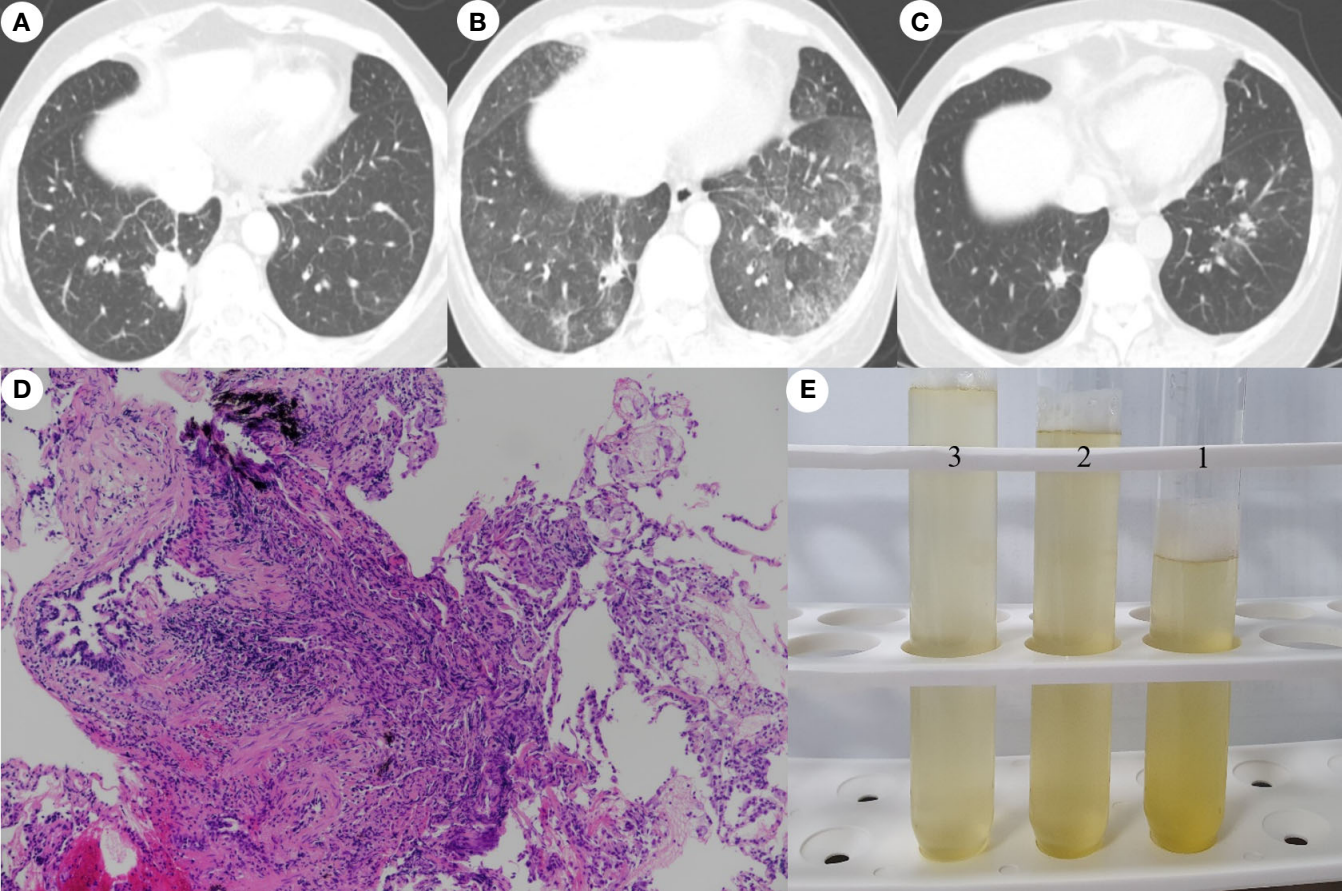
Chest computed tomography images showing. **(A)** A lobulated mass with pleural retraction in the posterobasal segment of the right lower lobe (RLL) and multiple tiny nodules in both lungs at the time of diagnosis. **(B)** Four months after initiating crizotinib, bilateral ground-glass opacities were observed, despite an obvious decrease in the size of the primary tumor in the RLL and multiple nodules in both lungs. **(C)** Nine weeks after crizotinib retreatment, a further decrease in the size of the primary RLL tumor and almost complete clearing of the bilateral ground-glass opacities was seen, without recurrence of ILD. **(D)** Transbronchial lung biopsy specimen showing patchy lymphocytic infiltration of the peribronchiolar interstitium and intra-alveolar accumulation of foamy macrophages (hematoxylin-eosin stain, x100). **(E)** Photograph of the non-diagnostic, yellowish fluid aspirated through bonchoalveolar lavage.

An otherwise healthy 54-year-old Chinese women residing in Korea, with no smoking history, presented with a 2-week history of a non-productive cough and palpable neck lymph nodes (LNs). Chest computed tomography (CT) showed a right lower lobe (RLL) mass with lung-to-lung metastases and pleural retraction (**Figure 1A**), and she was diagnosed with stage IV lung adenocarcinoma (cT4N3M1c) with two brain metastases. Mutational analysis of LN tissue revealed positivity only for ROS1 rearrangements, as determined by reverse transcription-polymerase chain reaction assay. Crizotinib was administered orally (250mg twice per day) as first-line chemotherapy following brain CyberKnife treatment. After 3 months, chest CT revealed good responses (beyond partial remission) of the RLL tumor, multiple LNs and lung-to-lung nodules. However, 4 months after initiating crizotinib, chest X-ray showed diffuse infiltration of both lower lobes. She exhibited dry cough and dyspnea on exertion. Physical examination revealed; respiratory rate of 18 beats/min, temperature 36.5°C, pulse of 90 beats/min, and blood pressure 130/80 mmHg. She had inspiratory crackles in both lung bases. The remainder of her examination was normal. Laboratory data included a white cell count of 5,050/mm^3^ (65.4% neutrophils, 0.1% eosinophils), an erythrocyte sedimentation rate of 15 mm/h (normal, 0-22 mm/h) and a C-reactive protein level of 0.5 mg/dL (normal, 0-0.5 mg/dL). Arterial blood gas values included a partial oxygen pressure of 73 mmHg, a partial carbon dioxide pressure of 35.9 mmHg, a pH of 7.42, and an oxygen saturation of 94% while resting on 2 L/min of oxygen. Our baseline target oxygen saturation range was 94–96%. Chest CT revealed newly developed extensive diffuse ground-glass opacities throughout both lungs, despite obvious shrinkage of the RLL primary tumor and multiple additional nodules (**Figure 1B**). Bronchoalveolar lavage (BAL) identified 94% lymphocytes, 2% monocytes and transbronchial lung biopsy demonstrated patchy lymphocytic infiltration of the peribronchiolar interstitium (**Figure 1D**). BAL was performed under local anesthesia and mild conscious sedation with midazolam according to the guidelines for the standardization of BAL, as described in our previous study (10). Echocardiography, laboratory tests, and BAL fluids (**Figure 1E**) showed no evidence of heart failure, rheumatologic diseases, pulmonary infections, or progression of lung cancer.

Taken together, we considered the lung lesions to be consistent with Grade 3 crizotinib-ILD according to the National Cancer Institute Common Terminology Criteria for Adverse Events (CTCAE) v5.0. and thus discontinued administration of crizotinib immediately. After withdrawing crizotinib, intravenous methylprednisolone (1 mg/kg daily for 5 days then switch to oral prednisolone 40mg/day, reduction dose by half dose every 4 weeks), empirical antibiotics, and prophylactic trimethoprim-sulfamethoxazole were initiated. She responded well to the corticosteroid therapy and the infiltration dramatically improved. The patient and her family expressed a desire to retry crizotinib therapy rather than conventional chemotherapy, even after the risk of recurrent ILD was explained. Therefore, and because there was no better alternative therapy for her lung cancer considering her preference and medical insurance, we restarted crizotinib (250 mg/day for 1 month and then 200 mg twice daily) 30 days after discontinuing the medication. The steroid dose has been gradually and successfully tapered and, at 9 weeks after crizotinib retreatment, there was neither disease progression nor recurrence of ILD (**Figure 1C**).

## Discussion

Over the past 20 years, various TKIs have been developed. These agents have dramatically improved patient survival and quality of life, and led to a paradigm shift in the treatment of various solid tumors (11). Crizotinib is an oral selective small-molecular TKI that suppress the activity of ALK and ROS1 kinases, as well as mesenchymal-epithelial transition (3). Crizotinib was initially approved for ALK-rearrangement NSCLC (12). Preclinical experiments have shown that cell lines with ROS1 rearrangement are highly sensitive to crizotinib (3). Also, in a clinical study of patients with advanced ROS1-rearrangement NSCLC, crizotinib showed marked antitumor activity, with an objective response rate of 72% and acceptable safety profile (1). Based on these results, the US FDA designated crizotinib as a breakthrough therapy for advanced ROS1-rearrangement NSCLC in 2016 (4). Crizotinib also provided meaningful clinical benefits and a durable response in East Asian patients with advanced ROS1-rearrangement NSCLC, with a similar objective response rate (71.7%) (2).

The findings of these clinical trials show that crizotinib has significant antitumor activity and a tolerable safety profile, with mild or moderate adverse events of visual disorders, diarrhea, nausea, and vomiting (1, 2, 13). As with other TKIs, however, the occurrence of crizotinib-ILD has been reported in the treatment of lung cancer (5–9, 14). Two systematic reviews of crizotinib-ILD in ALK-rearrangement NSCLC (15, 16), which included 4 PROFILE clinical trials and 29 studies, respectively, reported incidences of crizotinib-ILD of 1.2% (20/1669) and 1.8% (49/2706), respectively. The frequency of ILD was low with crizotinib compared to second-generation ALK-TKIs (alectinib, 2.6%; brigatinib, 7%), but the mortality rate reached 50%. Furthermore, the onset of ILD ranged from 3-763 days after initiation of crizotinib, indicating that ILD can occur at any time during therapy (15, 16). By contrast, crizotinib-ILD in patients with ROS1-rearrangement NSCLC has been reported only rarely (13, 17). In phase I/II studies of ROS1-rearrangement NSCLC, no case of crizotinib-ILD was reported (1, 2). Therefore, crizotinib-ILD in patients with ROS1-rearrangement NSCLC is very rare.

Although the exact mechanism underlying TKI-ILD has not yet been elucidated, one study suggested a putative mechanism, in which an epidermal growth factor receptor-TKI induces prolonged inflammation in the epithelial cells, which repair the airways, in turn, this may promote lung injury (18). A retrospective study suggested that crizotinib-ILD could be explained by drug-related hypersensitivity pneumonitis, which was associated with a longer response duration (19). However, more research is required to fully understand these issue.

Crizotinib-ILD is a relatively rare but sometimes fatal complication, and a major cause for permanent withdrawal of a drug during cancer treatment. Discontinuation of TKIs can be an especially significant clinical dilemma in the following cases: when a patient obtained marked clinical benefits from TKIs before ILD occurred and there was no suitable alternative therapy covered by their country’s medical insurance, and when rapid tumor progression is observed after discontinuing TKIs. In addition to crzotinib, entrectinib is the most recently an oral TKI approved by the US FDA (in August 2019) as a first-line therapy another option for patients with metastatic ROS1-rearrangement NSCLC (20). A recent comparative study of clinical trial results for crizotinib and entrectinib showed that the two TKIs have comparable or similar efficacy in ROS1-rearrangement NSCLC (20). In particular, entrectinib exhibited substantial systemic and intracranial efficacy in the first-line ROS1 TKI patients, and entrectinib-related pneumonitis were reported in less than 1% (1/134 patients) (21, 22). Unfortunately, our patient was unable to choose the entrectinib under current medical insurance.

**Figure 2:**
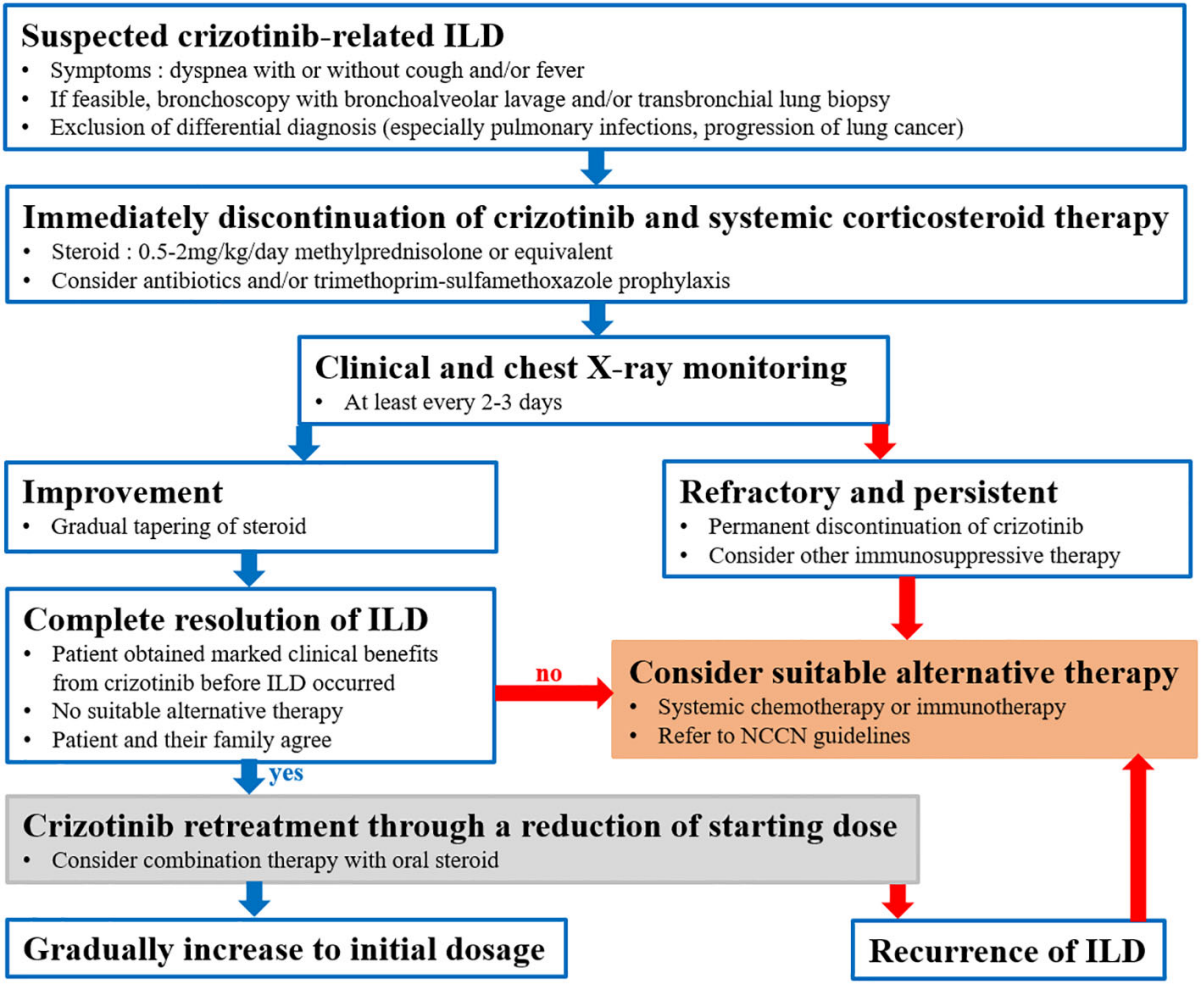
Suggested crizotinib retreatment strategy after crizotinib-related interstitial lung disease (ILD) in patients with NSCLC, based on a literature review.

As well as the present case, other cases of successful crizotinib retreatment after crizotinib-ILD have been reported (5–9). All of these cases are summarized in **Table 1**; ours was the only ROS1-rearrangement NSCLC case. All patients were Asian and used crizotinib as the first-line therapy. The median onset of ILD after the initiation of crizotinib was 6 weeks (range: 2-16 weeks), and the median time to restarting crizotinib after discontinuation was 5 weeks (range: 1-25 weeks). Except for one asymptomatic patient (8), all patients were treated with steroids, and combination therapy with steroids was considered when crizotinib was restarted. One patient suffered under conventional chemotherapy (9) and the remaining ones did not want to undergo chemotherapy as an alternative therapy. Generally, immediate discontinuation of the causative drug is recommended for symptomatic TKI-ILD. Systemic steroids are typically administered, although guidelines for TKI-ILD management have not been fully specified (23). Based on several cases and a literature review, we suggest an algorithm for a retreatment strategy to aid decision-making in situations where crizotinib retreatment should be considered in patients who develop crizotinib-ILD (**Figure 2**).

**Table 1:** Summary of cases of successful crizotinib retreatment after crizotinib-related interstitial lung disease (ILD) in patients with lung adenocarcinoma.

First author	Age (y)/sex smoker/ethnicity	Mutation stage/	Finding at the time of ILD occurrence				Crizotinib dose		Restart period^†^
			Symptoms	Onset^*^	Radiology	Steroid Tx	Initial	Re-Tx	
Yanagisawa (9)	53/female	ALK	Cough,	2 weeks	BL GGO	mPD^‡^	250 mg bid	250 mg bid	25 weeks
	no/Japanese	IV(M1c)	dyspnea	(14 days)		1000 mg			(6 months)^§^
Maka (6)	47/female	ALK	Cough,	8 weeks	BL GGO	PD^||^	250 mg qd	250 mg qd	8 weeks
	no/Indian	IV(M1c)	dyspnea	(2 months)		0.5 mg/kg		
Asai (8)	70/female	ALK	No	5 weeks	BL GGO	No	200 mg bid	250 mg qd§	4 weeks (2^nd^)^¶^
	no/Japanese	IV(M1c)		(35 days)					(28 days)
Tachihara (5)	70/male	ALK	Cough,	4 weeks	Lt GGO	PD^#^ 5 mg	250 mg bid	250 mg bid	1 week
	current/Japanese	IV(M1c)	fever	(25 days)				
Asai (7)	60/male	ALK	No	7 weeks	Lt GGO^**^	PD^††^	250 mg bid	250 mg 3T/W	6 weeks
	current/Japanese	IIIB	(50 days)	0.5 mg/kg				
Ryu	54/female	ROS1	Cough,	16 weeks	BL GGO	mPD	250 mg bid	250 mg qd	4 weeks
(present)	no/Chinese	IV(M1c)	dyspnea	(4 months)		1 mg/kg			(30 days)

^*^Time from initiation of crizotinib to onset of ILD; ^†^Time from discontinuation of crizotinib to retreatment; ^‡^Methylprednisolone (mPD) 1000mg daily for 3days, then the steroid dosage was tapered gradually on a weekly basis, until it was 2mg daily; ^§^Crizotinib retreatment after various conventional chemotherapies; ^||^Prednisolone at 0.5 mg/kg and gradually tapered over 8 weeks; ^¶^Crizotinib retreatment (250 mg qd) after 2 weeks of first ILD occurrence, but ILD recurrence at 45 days after crizotinib retreatment. second crizotinib retreatment at 28 days after ILD recurrence; ^#^Prednisolone (PD) (20 mg/day) use with initial crizotinib therapy due to multiple endobronchial metastases, and continued at 5 mg/day after reducing in a stepwise fashion. PD (20mg/day) used with crizotinib retreatment; ^**^Organizing pneumonia pattern confirmed by transbronchial lung biopsy; ^††^Prednisolone (PD) at 0.5 mg/kg and gradually tapered over 2 weeks; ALK, anaplastic lymphoma kinase; bid, twice daily; BL, bilateral; GGO, ground-glass opacity; Lt, left; qd, once daily; Re-Tx, retreatment; T/W, times per week; Tx, treatment.

In summary, to our knowledge, this is the first case report of successful crizotinib retreatment after crizotinib-ILD in a patient with ROS1-rearrangement NSCLC. There is no doubt that crizotinib plays a pivotal and irreplaceable role in the treatment of ALK- or ROS1-rearrangement NSCLC. Although crizotinib-ILD is a relative rare complication, it is sometimes fatal to patients and can pose a major clinical dilemma for clinicians. Therefore, crizotinib retreatment should be considered for closely monitored patients who develop crizotinib-ILD during lung cancer treatment if the following conditions are satisfied: the patient obtained marked clinical benefits from crizotinib before ILD occurred, there is no suitable alternative therapy covered by their country’s medical insurance or according to the patient’s preferences, and the ILD responded well to steroids.

## Data availability statement

The datasets for this article are not publicly available due to concerns regarding participant/patient anonymity. Requests to access the datasets should be directed to the corresponding author.

## Ethics statement

Ethical review and approval was not required for the study on human participants in accordance with the local legislation and institutional requirements. The patients/participants provided their written informed consent to participate in this study.

## Author contributions

H-SN treated the case and wrote the manuscript. WR wrote the manuscript and acquired clinical data. HC acquired clinical data. MP, JK, and J-SC participated in the diagnosis and treatment of the patients. LK and K-HL contributed to pathologic and radiologic response evaluation, respectively. All authors contributed to the article and approved the submitted version.

## Conflict of interest

The authors declare that the research was conducted in the absence of any commercial or financial relationships that could be construed as a potential conflict of interest.

## Publisher’s note

All claims expressed in this article are solely those of the authors and do not necessarily represent those of their affiliated organizations, or those of the publisher, the editors and the reviewers. Any product that may be evaluated in this article, or claim that may be made by its manufacturer, is not guaranteed or endorsed by the publisher.
